# Enhancing evidence-informed policymaking in medicine and healthcare: stakeholder involvement in the Commons Project for rare diseases in Japan

**DOI:** 10.1186/s40900-023-00515-5

**Published:** 2023-11-29

**Authors:** Atsushi Kogetsu, Moeko Isono, Tatsuki Aikyo, Junichi Furuta, Dai Goto, Nao Hamakawa, Michihiro Hide, Risa Hori, Noriko Ikeda, Keiko Inoi, Naomi Kawagoe, Tomoya Kubota, Shirou Manabe, Yasushi Matsumura, Koji Matsuyama, Tomoko Nakai, Ikuko Nakao, Yuki Saito, Midori Senoo, Masanori P. Takahashi, Toshihiro Takeda, Megumi Takei, Katsuto Tamai, Akio Tanaka, Yasuhiro Torashima, Yuya Tsuchida, Chisato Yamasaki, Beverley Anne Yamamoto, Kazuto Kato

**Affiliations:** 1 Department of Biomedical Ethics and Public Policy, Graduate School of Medicine, Osaka University, Suita, Japan; 2 School of Medicine, Hiroshima University, Hiroshima, Japan; 3 Department of Medical Informatics and Management, Institute of Medicine, University of Tsukuba, Tsukuba, Ibaraki Japan; 4 Department of Stem Cell Therapy Science, Graduate School of Medicine, Osaka University, Suita, Osaka Japan; 5 Department of Dermatology, Graduate School of Biomedical and Health Sciences, Hiroshima University, Hiroshima, Japan; 6 Japanese Society of Tuberous Sclerosis Complex Family Net Committee, Yokohama, Kanagawa Japan; 7 Department of Dermatology, Hiroshima Citizens Hospital, Hiroshima, Japan; 8 Commons Project, Osaka University, Suita, Osaka Japan; 9 NPO Japan Marfan Association, Kuwana, Mie Japan; 10 MECP2 Duplication Syndrome Family Association, Suita, Osaka Japan; 11 Department of Clinical Laboratory and Biomedical Sciences, Graduate School of Medicine, Osaka University, Suita, Osaka Japan; 12 Department of Transformative System for Medical Information, Graduate School of Medicine, Osaka University, Suita, Osaka Japan; 13 Osaka National Hospital, Osaka, Japan; 14 Department of Medical Informatics, Graduate School of Medicine, Osaka University, Suita, Osaka Japan; 15 Japanese Huntington’s Disease Network (JHDN), Tokyo, Japan; 16 Shirasagi-aiai-kai, Himeji, Hyogo Japan; 17 NPO Myotonic Dystrophy Patients’ Group of Japan (DM-Family), Tokyo, Japan; 18 Department of Surgery, Nagasaki University Graduate School of Biomedical Sciences, Nagasaki, Japan; 19 Center for Intractable Diseases and ImmunoGenomics (CiDIC), Health and Nutrition (NIBIOHN), National Institutes of Biomedical Innovation, Ibaraki, Osaka Japan; 20 HAEJ, Non-Profit Patient Organization for Hereditary Angioedema in Japan, Kakogawa, Hyogo Japan; 21 HAEi, Non-Profit International Patient Organization for Hereditary Angioedema Registered in the US, Fairfax City, VA USA; 22 Graduate School of Human Sciences, Osaka University, Suita, Osaka Japan

**Keywords:** Rare-disease policy, Priority setting, Stakeholder involvement, Patient involvement, Patient and public involvement (PPI), Evidence generation, Evidence-based policymaking (EBPM), Evidence-informed policymaking (EIPM), Quality of life (QOL)

## Abstract

**Background:**

Although stakeholder involvement in policymaking is attracting attention in the fields of medicine and healthcare, a practical methodology has not yet been established. Rare-disease policy, specifically research priority setting for the allocation of limited research resources, is an area where evidence generation through stakeholder involvement is expected to be effective. We generated evidence for rare-disease policymaking through stakeholder involvement and explored effective collaboration among stakeholders.

**Methods:**

We constructed a space called ‘Evidence-generating Commons’, where patients, family members, researchers, and former policymakers can share their knowledge and experiences and engage in continual deliberations on evidence generation. Ten rare diseases were consequently represented. In the ‘Commons’, 25 consecutive workshops were held predominantly online, from 2019 to 2021. These workshops focused on (1) clarification of difficulties faced by rare-disease patients, (2) development and selection of criteria for priority setting, and (3) priority setting through the application of the criteria. For the first step, an on-site workshop using sticky notes was held. The data were analysed based on KJ method. For the second and third steps, workshops on specific themes were held to build consensus. The workshop agendas and methods were modified based on participants’ feedback.

**Results:**

The ‘Commons’ was established with 43 participants, resulting in positive effects such as capacity building, opportunities for interactions, mutual understanding, and empathy among the participants. The difficulties faced by patients with rare diseases were classified into 10 categories. Seven research topics were identified as priority issues to be addressed including ‘impediments to daily life’, ‘financial burden’, ‘anxiety’, and ‘burden of hospital visits’. This was performed by synthesising the results of the application of the two criteria that were particularly important to strengthen future research on rare diseases. We also clarified high-priority research topics by using criteria valued more by patients and family members than by researchers and former policymakers, and criteria with specific perspectives.

**Conclusion:**

We generated evidence for policymaking in the field of rare diseases. This study’s insights into stakeholder involvement can enhance evidence-informed policymaking. We engaged in comprehensive discussions with policymakers regarding policy implementation and planned analysis of the participants’ experiences in this project.

**Supplementary Information:**

The online version contains supplementary material available at 10.1186/s40900-023-00515-5.

## Abstract (plain language summary)

Stakeholder involvement is significant for effective policymaking in the field of rare diseases. However, practical methods for this involvement have not yet been established. Therefore, we developed the ‘Commons project’ to generate valuable policymaking information and explore effective ways for stakeholders’ collaboration. This article explains the process and results of 25 continuous workshops, held from 2019 to 2021 with 43 participants, including patients, family members, researchers, and former policymakers. The main achievements of the discussion that took place in the ‘Commons’ included a presentation of the overview of the difficulties faced by patients with rare diseases and formulation of high priority research topics.

First, the difficulties faced by patients with rare diseases were grouped into 10 categories. Second, seven research topics were identified as priority issues including ‘impediments to daily life’, ‘financial burden’, ‘anxiety’, and ‘burden of hospital visits’. During the project process, positive effects such as capacity building, opportunities for interactions, mutual understanding, and empathy among the participants, were identified. Beyond the context of the field of rare diseases and science of policy, these findings are useful for the future of society, including co-creation among stakeholders and patient and public involvement. Based on this study’s results, we have initiated communications with policy stakeholders in the field of rare diseases, with the aim of policy implementation.

**Supplementary Information:**

The online version contains supplementary material available at 10.1186/s40900-023-00515-5.

## Background

### Evidence generation through stakeholder involvement in policy making

In recent years, the roles of patients and their families have changed dramatically in medical practice, medical research, drug discovery, and medical device development [1–3]. In medical research, the role of patients has changed from ‘research participants’ to ‘experts with lived experience’, with some being involved in various research processes, such as study planning and design, recruitment of participants, analysis and interpretation of results, and the dissemination of research findings [4]. Patient involvement is prominent in Europe and USA, and several initiatives have recently been reported in Japan [5–9]. Organisations that play an important role in national policy, such as the Japan Agency for Medical Research and Development (AMED) and the Pharmaceuticals and Medical Devices Agency (PMDA), have also demonstrated their commitment to promoting patient involvement [10, 11].

In the field of medicine and healthcare, the ways of policy development (including a wide range of topics, from the design of medical and insurance systems to medical research, education, and information dissemination) are being explored in many countries. In the context of policymaking, patient involvement has attracted increasing attention [12]. Policies cover diverse targets; however, policies on priority setting for research and development are essential [13, 14]. Research priority setting for the allocation of research resources is placed upstream of the research cycle, and thus patient involvement in this process is particularly crucial [12, 15]. However, a consensus around the best methods for patient involvement in setting priorities is yet to be established.

To effectively allocate limited resources, they should be prioritised. From the perspective of evidence-informed policymaking (EIPM) which has recently gained recognition, this priority setting should be based on evidence [16]. However, no settled agreement exists on what constitutes ‘evidence for policies’; this is an ongoing debate [17, 18]. In evidence-based medicine (EBM), randomised controlled trials (RCTs) are the gold standard for evaluating the effects of treatments. Nevertheless, in this classical hierarchy of evidence making, other methods, including qualitative studies, are also often used before the design of a RCT study [19]. In EIPM, such ideas are sometimes discussed as a reference; however, in policy discussions, it is increasingly recognised that evidence can come from a variety of sources, including not only quantitative data, but also qualitative data from individuals’ experiences, minutes of government committees and parliaments, and expert opinions [20]. Based on these arguments, this study tentatively defines evidence as *information that can be referred to when forming a policy*.

Although no consensus exists on the definition of evidence, Cash et al*.* and Parkhurst examined the characteristics of information that can be evidence for policymaking from several perspectives [21, 22]. They identify the attributes of information for policymaking, namely credibility (being scientifically reliable), salience (being relevant to decision-makers’ needs), and legitimacy (being fair in the information-producing process and respecting stakeholders’ diverse values, concerns, and perspectives). Furthermore, when policy decisions are made based on scientific information, various stakeholders involved in the decision-making process uniquely recognise and evaluate these three attributes. Moreover, these attributes have ‘thresholds’, and the information used in policy-making is effective when it simultaneously satisfies reliability, salience, and legitimacy for multiple audiences [21].

Based on this concept, Katirai et al*.* pointed out that patients, as concerned individuals, can generate better evidence for all three attributes by being involved in the evidence generation process through structured mechanisms [12]. While these activities do not generate quantifiable information, they are gradually gaining acceptance for generating evidence for policymaking and can serve as the effective methods for EIPM [23–25]. Specifically, Priority Setting Partnerships (PSPs) by the James Lind Alliance (JLA) and the Child Health and Nutrition Research Initiative (CHNRI) attempt to set research priorities as evidence with stakeholders, including patients [26, 27]. However, these activities are still at the exploratory stage of policy implementation, and comprehensively, methods to generate evidence through the involvement of stakeholders, including patients, citizens, healthcare providers, and other experts in various fields, have not been fully established [28]. In deliberative democracy, methods to involve stakeholders are present. These include citizen’s panels, consensus conferences deliberative public polls, and participatory budgeting [29–31]. However, in the fields of medicine and healthcare, methods in deliberative democracy are limited to discussions on biobanks [32], and their feasibility and effectiveness for policymaking have not been fully clarified. Additionally, according to Staley et al., frameworks for promoting and evaluating patient involvement are context-dependent and not simply generalisable; therefore, practices and descriptions of these—contexts, mechanisms, and outcomes—need to be accumulated [28].

### Policies in the field of rare diseases in Japan

Rare-disease policy, which has been prioritised in many countries in recent years, appears to be one area where evidence generation through stakeholder involvement, as discussed above, is expected to be effective.

Though the definition of rare disease varies from country to country, it refers to diseases that exhibit a certain prevalence (e.g. less than 1 in 1600 in the US and less than 1 in 2000 in the EU). More than 6000–7000 diseases have currently been identified, with the total number reaching up to 1.5–6.2% of the population worldwide [33]. Many of these diseases are chronic and lack curative treatments, leading not just to significant physical burden but also psychological burden for the patients. In addition, there are also financial and social burdens on patients and their families to maintain their daily lives [34–38]. Notably, a variety of rare diseases share this disease burden [34, 36]. However, to our knowledge, few attempts have been made to provide an overview of this common disease burden. Furthermore, few attempts have been made to link this to policymaking.

In Japan, systems for rare diseases have been built within the framework of ‘Nambyo (intractable diseases)’ since the 1970s [39]. In 2014, the Act on Medical Care for Patients with Intractable Diseases was passed, and in January 2015, 110 diseases became eligible for medical expense subsidies, which have now been further expanded to 338 diseases [40]. Movements led by patient groups have influenced the process of enacting the law [41]. However, while these movements are arguably important, they could also simply result in conveying the opinions of patients, their families, and patient advocacy groups to policymakers in a petition-type manner. Hence, conveying patients’ views as evidence that can be used in policymaking is important, as mentioned above. Additionally, to reflect on the perspectives and opinions of patients with diseases for which no patient groups exist or for which patient groups are inactive, and the stakeholders other than patients in policy will be a challenge in the future. In Japan, stakeholder involvement in these policy areas has been insufficient.

### Objectives of the study

Against this background, we designed the Commons Project with two objectives: (1) to generate evidence that contributes to policymaking in the fields of medicine and healthcare, particularly in the field of rare diseases and (2) to explore, through practice, effective stakeholder involvement and, particularly, specific ways of collaboration for such evidence generation. Here, the ‘Commons’ refers to a place where patients, family members, researchers, and policymakers can share their knowledge and experiences, and engage in continuous deliberations on evidence generation, with the ‘living social system of creative agents’, which consists of shared resources and the communities that manage them, by devising their own rules, traditions, and values, as argued by Bollier [42]. According to his theory, the commons ‘is primarily about the social practices of commoning—acts of mutual support, conflict, negotiation, communication and experimentation that are needed to create systems to manage shared resources’, which is the theoretical basis of the concept of our ‘Commons’. The ‘Commons’ has been named the ‘Evidence-generating Commons’ (hereafter simply the ‘Commons’). In this paper, we report the research activities of the Commons project, specifically the processes and results of the clarification of various kinds of difficulties faced by patients with rare diseases and their families and the priority setting when addressing these difficulties. Furthermore, based on the results of this study, we discuss the implications of the methods used and quality of the evidence generated.

## Methods

### Overview of the methods of this study

#### Recruitment and characteristics of the participants

Participants were recruited from patients with rare diseases and family members, and each gave consent for participation in the research. For the recruitment, the research team’s networks from the previous/ongoing studies conducted by us were utilised, including RUDY JAPAN [5], which is a research project targeting multiple rare diseases in partnership with patients. Participants were also contacted individually by researchers, based on personal connections and introductions. Patients and family members representing 10 disease areas were recruited (Box 1). These diseases share the common characteristics of being hereditary, having no curative treatment, and having a long-term disease burden. Many participants were involved in the activities involving patient groups. Further, experts in medical research on neuromuscular diseases, hereditary angioedema, and epidermolysis bullosa, and those in social sciences, research governance, ethics, and medical information were involved in the project. The project also involved former policymakers who had worked in government organisations, such as the Ministry of Health, Labour and Welfare, and AMED.

**Box 1:** Disease areas involved

< Diseases covered at the start of the project>
Myotonic dystrophy
Skeletal muscle channelopathies (non-dystrophic myotonias)
Hereditary angioedema
MECP2 duplication syndrome
Huntington’s disease
Spinocerebellar degeneration
Tuberous sclerosis
Marfan’s syndrome
<Diseases added to the list during the study>
Epidermolysis bullosa
Retinitis pigmentosa

#### Design of the workshops

As described above, several methods for priority setting with relevant stakeholders have been reported. However, we did not know whether methods used in other countries were appropriate for Japanese society. We also did not have an established methodology to train participants. For these reasons, we decided to develop a new method to set a research priority in the rare-disease area.

**Fig. 1:**
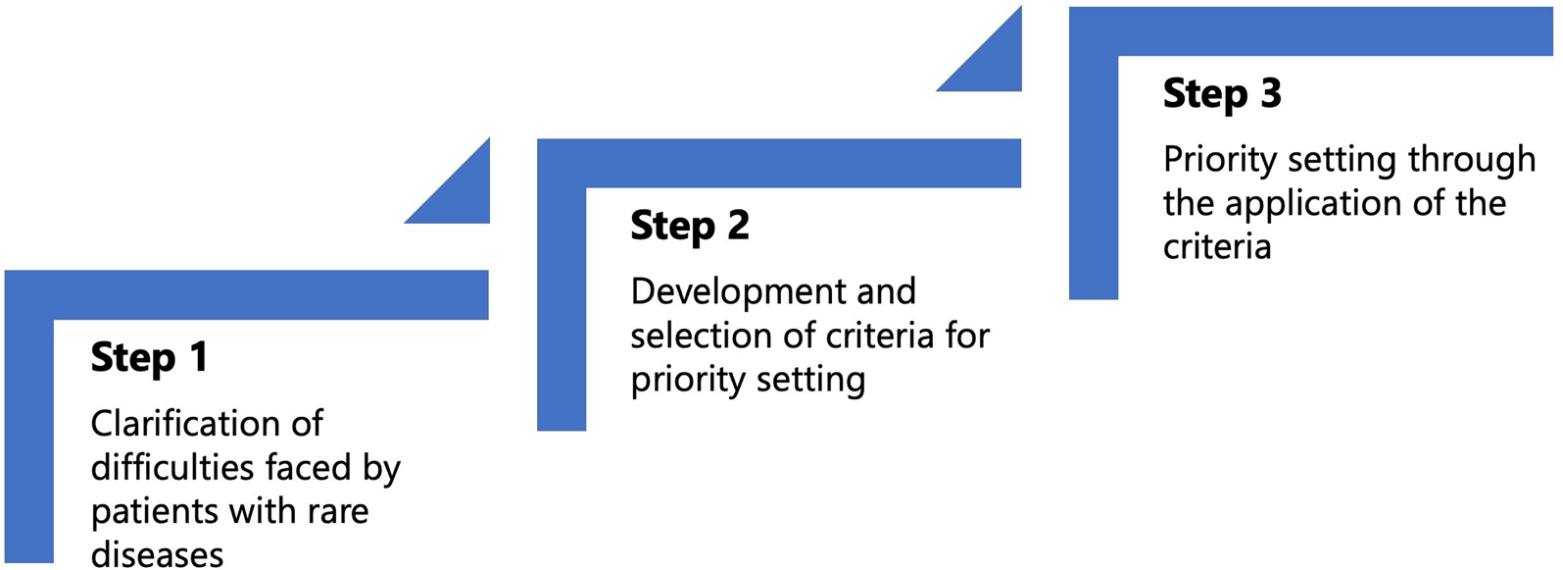
Three steps of priority setting were undertaken in the study

Consecutive workshops were held in the ‘Commons’, with participants discussing the themes set out in each workshop. To respect stakeholders’ diverse values and beliefs and ensure fairness in the treatment of views and interests throughout the project, the participants were asked not only to think about the disease with which they had a first-hand experience but also to think beyond their disease area. The researchers specialising in research ethics and governance (AK, MI, TA, NH, KK) acted as facilitators in the workshops. The main facilitators (AK and KK) explained the themes and discussion points at the beginning of the workshops and participants shared their opinions on each theme. Individual thinking time was provided before all group work, and each participant was given the opportunity to speak. In workshops where consensus was required, the facilitators proposed a consensus draft, based on participants’ opinions. If the participants disagreed on the same point, an amendment was proposed based on the differing opinions. This process was repeated until a final agreement was reached. The findings were also synthesised, based on participant consensus. According to the final agreement in each workshop, the facilitator prepared a proposal for the integrated findings. The participants shared their opinions on the proposal and decided on the final results. Workshop themes were broadly divided into three steps for priority setting (Fig. 1): (1) clarifying the difficulties faced by patients with rare diseases and their families, (2) developing and selecting criteria for priority setting, and (3) setting priorities by applying the criteria. The following section provides details. The starting point for the research priority setting was to map the difficulties faced by patients and their families by considering issues closer to the patients. Furthermore, we developed and selected decision criteria to ensure a consistent approach to setting priorities among a diverse group of individuals, as using different criteria based on standpoints would hinder meaningful discussions.

We employed a reflexive approach by which themes and discussion points were modified as we went along to reflect the opinions and ideas that had emerged in previous meetings. Additionally, the setting of research priorities by considering the difficulties faced by patients and their families as topics of academic research in the fields of medicine and healthcare from Step 2 onwards was not fully understood by the participants, and the discussion often deviated to policies and systems. Therefore, we devised a way to deepen the understanding that this study focuses on research as problem-solving by considering specific research questions in several research topics (Additional step). Furthermore, we introduced a short talk to clarify the breadth of the field of ‘academic research’ in response to participants’ diverse understandings of the term ‘research’ and the tendency to equate it with clinical trials or clinical research.

### Step 1: Clarification of difficulties faced by patients with rare diseases

An on-site workshop was held for all the stakeholders in March 2019. A total of 28 participants, including 13 patients and family members from 7 disease areas, 12 researchers, and 3 former policymakers, attended the workshop. The workshop was designed to address the question ‘What are the difficulties faced by patients with rare diseases?’ and ‘What can we do to address them?’ The method was based on the ‘Opinion Eliciting Workshops’ proposed by Yagi and Nakagawa [43] (see Additional file 1: Programme of the on-site workshop).

First, the participants were given 10 min to write down the difficulties of rare disease patients on yellow sticky notes and solutions on red sticky notes, and for the next 40 min, sticky notes with similar content were collected and organised as part of a group work. Subsequently, the participants were asked to write down solutions that they proposed following the previous group work on blue sticky notes for five minutes, and finally, the group organised and discussed them once more.

After the on-site workshop, as a first step of the analysis, each sticky note was coded and categorised based on the KJ method [44, 45] such that the difficulties faced by patients with rare diseases were sorted out in groups. Next, an online workshop was held in November 2019. A total of 19 participants (5 patients or family members, 12 researchers, and 2 former policymakers) attended the workshop to discuss ways to summarise the results of the analysis.

### Step 2: Development and selection of criteria for priority setting

Three online workshops were organised between December 2019 and January 2020 (each with 8 to 12 participants) to discuss the criteria to set priorities of the research topics when the extracted items about ‘difficulties faced by patients with rare diseases’ were considered as ‘research topics’. Prior to this, the participants were asked for the drafts of the ‘criteria for priority setting’. Based on the results, opinions were exchanged in an online workshop to develop candidate criteria.

The participants were then polled using an online form to select 10 candidate criteria that they considered particularly important. The results were used to determine the criteria that should be prioritised for application in workshops. The decision on the selection criteria was made by all workshop participants.

### Step 3: Priority setting through the application of the criteria

Seven online workshops were organised between January 2020 and January 2021 (each with 6–18 participants). Discussions were held on the application of the eight criteria for priority setting selected in Step 2 to set priorities for research topics which were clarified as difficulties faced by patients with rare diseases. During the workshops, each participant first selected 5 to 10 research topics that fitted the criteria. Subsequently, following a discussion, they classified the topics into three categories: ‘good fit’, ‘moderate fit’, and ‘poor fit’ (some of the criteria were classified into four levels: ‘good fit’, ‘moderate fit’, ‘slight fit’, and ‘poor fit’). The workshops were conducted with all the participants in one group when the number of participants was small, whereas the discussions were broken down into several groups when there were many participants.

### Additional step: brainstorming session on specific research questions

In two online workshops held in January 2021, two research topics identified as ‘research topics with high priority’ based on the results of the previous discussions were selected for brainstorming on specific research questions on research topics. The primary purpose of this step was to deepen participants’ understanding with regard to the focus of this study on research as problem-solving. The workshops were conducted using the online sticky note tool Apisnote [46], in which each participant proposed a research question related to the research topic. In this session, the participants were asked to present their ideas freely without limiting the scope of the research question or considering the feasibility of the research.

## Results

### Establishment of the ‘Commons’

At the beginning of the study, 31 individuals initially participated in the ‘Commons’, including 14 patients with any of 8 diseases or family members, 14 researchers (including 7 clinicians), and 3 former policymakers.

During the three-year research period, 25 workshops were held, including the four research steps described in the Methods section and those for reviewing previous study findings and considering subsequent directions (Table 1).

**Table 1:** The number of participants in each step and of meetings for the review

Step	Aim of each workshops/poll/meetings	Date	Methods	Number of participants		
				Patients and family members	Researchers	Former policymakers
Step1	To share the aim of the project and experiences of the patients	6th Mar 2019	Workshop (online)	7	6	2
	To understand the difficulties faced by patients with rare diseases	9th Mar 2019	Workshop (on-site)	13	12	3
	To better summarise the results of the analysis	19th Nov 2019	Workshop (online)	5	12	2
Step2	To develop the candidate criteria to set priorities for the research topics	23rd Dec 2019, 27th Dec 2019, 7th Jan 2020	Workshop (online)	8	10	2
	To select 10 candidate criteria	From 10 to 17th Jan 2020	Poll (online)	12	7	2
Step3	To determine the 5 criteria to apply	17th Jan 2020	Workshop (online)	3	3	0
	To apply the selected criteria to set priorities for research topics					
	Criterion (1) ‘Research topics related to various QOL aspects, such as psychological and lifestyle aspects’	17th Jan 2020	Workshop (online)	3	3	0
	Criterion (2) ‘Research topics related to expected findings meant to alleviate patients’ pain and burden and lead them to gain “independence”’	18th Jan 2020	Workshop (online)	4	4	0
	Criterion (3) ‘Research topics that patients readily experience as issues and would directly feel the benefits of, if properly addressed’ and Criterion (4) ‘Topics on which research has been set aside or delayed because the number of the patients with the rare disease is so small, or the diseases were not life-threatening’	28th Jan 2020	Workshop (online)	3	7	1
	Criterion (8) Research topics where Internet use is expected to be an effective problem-solving tool	14th Sep 2020	Workshop (online)	6	10	0
	Criterion (7) Research topics related to children	18th Jan 2021	Workshop (online)	8	7	1
	Criterion (5) Research topics that affect the surrounding environment, such as family and healthcare providers	19th Jan 2021	Workshop (online)	3	7	1
	Criterion (6) Research topics on problems that cannot be resolved even though patients have been making their own efforts	21st Jan 2021	Workshop (online)	7	8	1
Additional step	To brainstorm on specific research questions					
	Research topic: ‘Anxiety’	7th Jan 2021	Workshop (online)	5	11	1
	Research topic: ‘Impediments to daily life’	9th Jan 2021	Workshop (online)	4	7	1
Reviewing	To review previous study findings and considering subsequent directions	30th May 2020	Meeting (online)	7	12	1
		15th Apr 2021	Meeting (online)	8	9	1

In the process, new members were added to the ‘Commons’ through the network of RUDY JAPAN and the introduction of a participant to the ‘Commons’. Eventually, the ‘Commons’ consisted of 43 participants: 21 patients with any of the 10 diseases or family members, 17 researchers (including 9 clinicians), and 5 former policymakers.

Throughout the study, the ‘Commons’ positively affected the participants. All the project participants experienced mutual learning and personal growth, and trust was fostered through a friendly atmosphere of interaction among patients with common concerns as well as between patients and researchers. Specifically, some comments from the patients included: ‘It allowed me to think not only of my disease but also about patients with other diseases, which broadened my perspective’ and; ‘I was surprised at how many researchers in the world are willing to pick up patients’ voices’. Conversely, researchers’ comments included: ‘I realised that I had no idea what difficulties patients faced in their daily lives’ and; ‘I realised the difficulty explaining things in an easy-to-understand language that is not jargon’.

### Difficulties faced by patients with rare diseases

A total of 31 items emerged after 228 sticky notes were coded with the ‘difficulties faced by patients with rare diseases’ presented at the on-site workshop. The results showed that patients with rare diseases faced highly diverse issues, not only limited to healthcare but also related to their daily lives, families, and social issues. Thus, we grouped the 31 items into 10 categories, such as ‘lifestyle issues’, ‘family’, ‘social issues’, and ‘recognition/understanding’.

**Fig. 2:**
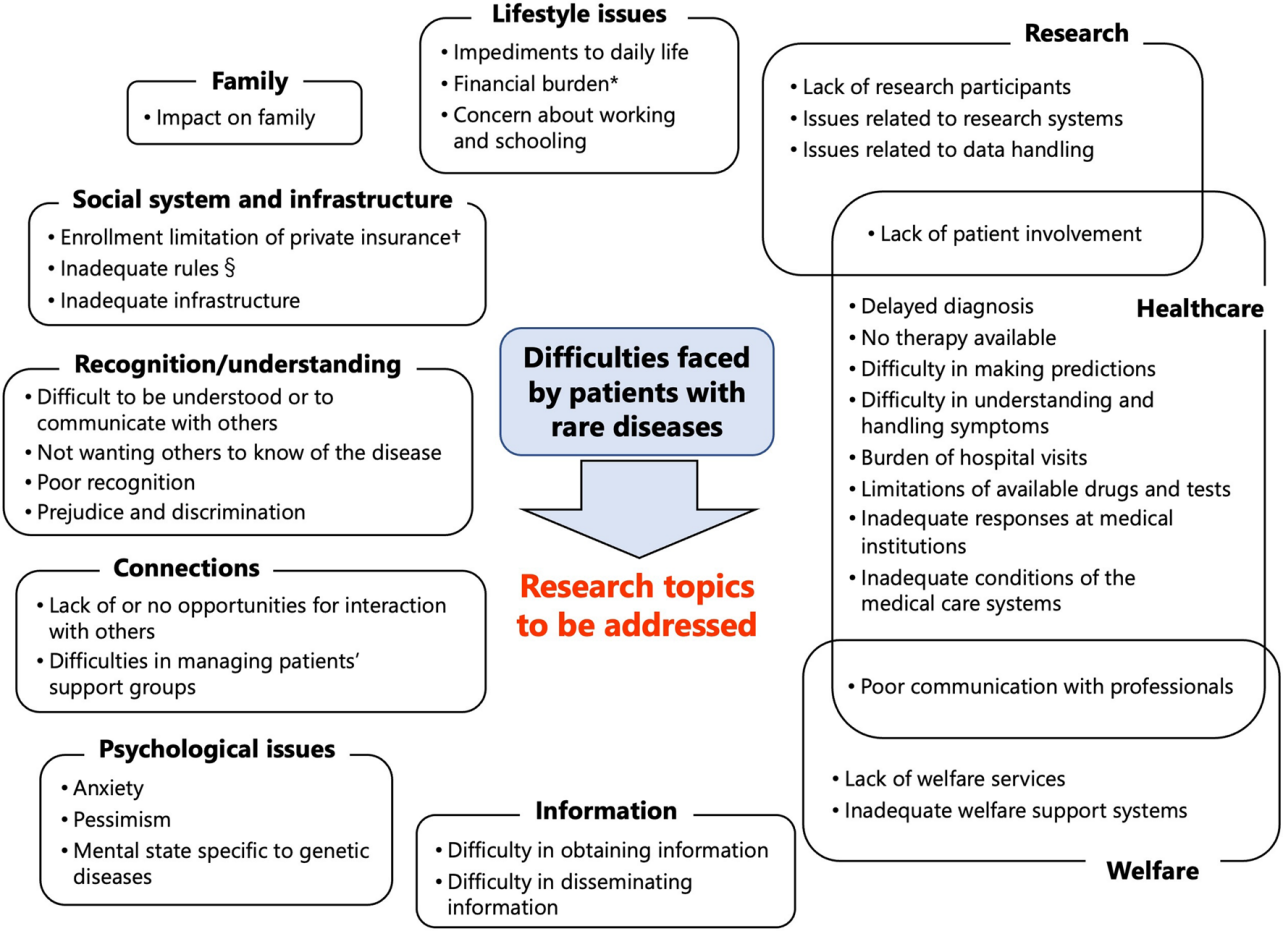
The overall picture of the ‘difficulties faced by patients with rare diseases’. *There are financial burdens, including medical expenses, and restrictions on subsidies based on the severity of the diseases and household income. †In Japan, in addition to the universal health insurance system, some people take out private life insurance and medical insurance. §Inadequate rules and regulations make it difficult to decide, for example, whether or not to inform the airline about their diseases when boarding an aircraft

Based on the results of this analysis, the participants of the ‘Commons’ discussed how the overall picture should be organised. This led to general agreement on grouping by categories and items. However, changes have been proposed for some wording and grouping. Specifically, the category originally described as ‘social issues’ was changed to ‘social systems and infrastructure’. Furthermore, a suggestion was made to move the item labelled ‘prejudice and discrimination’, which was originally categorised under ‘social issues’, to ‘recognition/understanding’. Consequently, the initial 31 items increased to 33. An overall picture is shown in Fig. 2.

### Selected criteria for priority setting

Following the workshop discussions, 22 criteria for priority setting were proposed, such as ‘research topics related to life and death’, ‘those related to physical function’, ‘those related to various quality of life (QOL) aspects such as psychological and lifestyle aspects’, ‘those related to many rare diseases’, and ‘those specific to rare diseases’. A total of 21 participants responded to the subsequent voting, including 12 patients or family members, 7 researchers, and 2 former policymakers. The results are presented in Table 2. Many votes were cast for criteria such as ‘research topics related to various QOL aspects such as psychological and lifestyle aspects’, ‘research topics related to life and death’, and ‘research topics related to expected research findings meant to alleviate the pain and burden of patients and lead them to gain “independence”’. The three criteria that did not apply to voting were additional ones that were proposed during voting.

**Table 2:** Results of voting on ‘selecting 10 criteria considered to be particularly important’

Candidates of criteria for priority setting	Total (21)	Patients and family members (12)	Researchers (7)	Former policymakers (2)
Research topics related to various QOL aspects such as psychological and lifestyle aspects	18	11	5	2
Research topics related to life and death	17	9	6	2
Research topics related to expected findings meant to alleviate patients’ pain and burden and lead them to gain ‘independence’	16	11	4	1
Research topics that cannot be solved without involving researchers or policymakers	13	9	4	0
Research topics that patients readily experience as issues and would directly feel the benefits of, if properly addressed	13	7	5	1
Research topics related to physical function	11	6	3	2
Research topics that directly affect the diseases or the patients themselves	11	8	3	0
Research topics that impact society as a whole (social systems or individuals’ behaviours)	11	4	6	1
Research topics on the difficulties that cause other problems, and their resolution which is expected to simultaneously solve many other problems	10	4	5	1
Research topics related to children	9	6	2	1
Research topics related to many rare diseases	8	4	3	1
Research topics specific to rare diseases	8	4	3	1
Research topics whose findings could have a ripple effect beyond rare intractable diseases	8	4	2	2
Research topics that have big disadvantages before and big advantages after finding a solution	8	5	2	1
Research topics related to expected research findings that lead to reducing the burden imposed on families and healthcare providers	8	6	1	1
Topics of research in which resources and knowledge are already available, and their application remains a bottleneck	8	5	3	0
Research topics with few studies addressing the issue	8	4	4	0
Research topics where Internet use is expected to be an effective problem-solving tool	7	4	2	1
Research topics that affect the surrounding environment, such as families and healthcare providers	6	5	1	0
Research topics expected to have a high cost-effectiveness	5	1	2	2
Research topics on problems that cannot be resolved even though patients have been making their own efforts	5	4	1	0
Topics of research that can be carried out with limited time and resources	4	1	3	0
Topics on which research has been set aside or delayed because the number of the patients with the rare disease is so small, or the diseases were not life-threatening	N/A	N/A	N/A	N/A
Research topics that require legal knowledge and that aim to bridge gaps between real life and related laws	N/A	N/A	N/A	N/A
Research topics that aim to develop new applications and methods for newly discovered or invented technologies (e.g. iPS cells)	N/A	N/A	N/A	N/A

During the discussion, an analysis of the results of voting by each stakeholder was suggested. The results showed that the voting rate by the patients tended to be higher than that by the researcher side for criteria such as ‘research topics related to expected findings that lead to reducing the burden imposed on families and healthcare providers’ and ‘research topics that affect the surrounding environment, such as families and healthcare providers’.

In the workshops held after these results were presented, it was first decided to select some criteria from the perspective that are particularly important for strengthening future research on rare diseases. Priority was given to those criteria that received the highest percentage of votes in the preliminary questionnaire, but the following two were not selected at that time for their respective reasons: the criterion ‘research topics related to life and death’, was considered to have already been given some priority in research and the criterion ‘research topics that cannot be solved without involving researchers or policymakers’ was considered to be more an approach to solving the problem than a criterion for selecting the research topic to be addressed. Three additional criteria added during the voting were also included in the discussion. Of these, the criterion ‘topics on which research has been set aside or delayed because the number of the patients with rare disease is so small, or the diseases were not life-threatening’ was deemed particularly important by the participants; therefore, we decided to select it.

The criteria selected based on these discussions are presented in Box 2. Four criteria were selected as ‘criteria that are particularly important for strengthening future research on rare diseases’: (1) ‘research topics related to various QOL aspects, such as psychological and lifestyle aspects’, (2) ‘research topics related to expected findings meant to alleviate patients’ pain and burden and lead them to gain “independence”’, (3)‘research topics that patients readily experience as issues and would directly feel the benefits of, if properly addressed’ and (4) ‘topics on which research has been set aside or delayed because the number of the patients with the rare disease is so small, or the diseases were not life-threatening’.

**Box 2:** The criteria selected

Category 1: Criteria that are particularly important for strengthening future research on rare diseases
(1) Research topics related to various QOL aspects, such as psychological and lifestyle aspects
(2) Research topics related to expected findings meant to alleviate patients’ pain and burden and lead them to gain ‘independence’
(3) Research topics that patients readily experience as issues and would directly feel the benefits of, if properly addressed
(4) Topics on which research has been set aside or delayed because the number of the patients with the rare disease is so small, or the diseases were not life-threatening
Category 2: Criteria in which patients and family members voted more than researchers and former policymakers
(5) Research topics that affect the surrounding environment, such as family and healthcare providers
(6) Research topics on problems that cannot be resolved even though patients have been making their own efforts
Category 3: Criteria with specific important perspectives that were not covered by the criteria in Categories 1 and 2
(7) Research topics related to children
(8) Research topics where Internet use is expected to be an effective problem-solving tool

Subsequently, as a characteristic of the ‘Commons’ is the participation of different stakeholders, it was decided to select the criteria for which the patients and family members voted more than the researchers and former policymakers. After discussion on the three criteria that accounted for more than 70% of the total number of votes cast by the patient side, a decision was made to select criterion 5, ‘research topics that affect the surrounding environment, such as family and healthcare providers’, and criterion 6, ‘research topics on problems that cannot be resolved even though patients have been making their own efforts’. The decision was made not to select the other one, ‘research topics about which expected findings lead to reducing the burden on patients’ families and healthcare providers’, as it might be included in criterion 5.

Furthermore, as the aforementioned criteria were selected from perspectives with which many participants agreed, we considered that some important perspectives were not covered solely by them. However, as it was impossible to incorporate them all, the workshop participants agreed that two of the most important criteria would be selected, namely criterion 7, ‘research topics related to children’ and criterion 8, ‘research topics where Internet use is expected to be an effective problem-solving tool’.

### Results of priority setting through the application of criteria

The results of the application of the criteria selected in Step 2 are presented below. Notably, for criteria 3, 4, and 6, which were not applicable, conclusions could not be drawn during the actual application of the criteria because all research topics had a good fit to the same extent (criterion 4) or because of too wide a range of interpretation among the workshop participants (criteria 3 and 6); therefore, these criteria were not suitable for setting priorities.

**Fig. 3:**
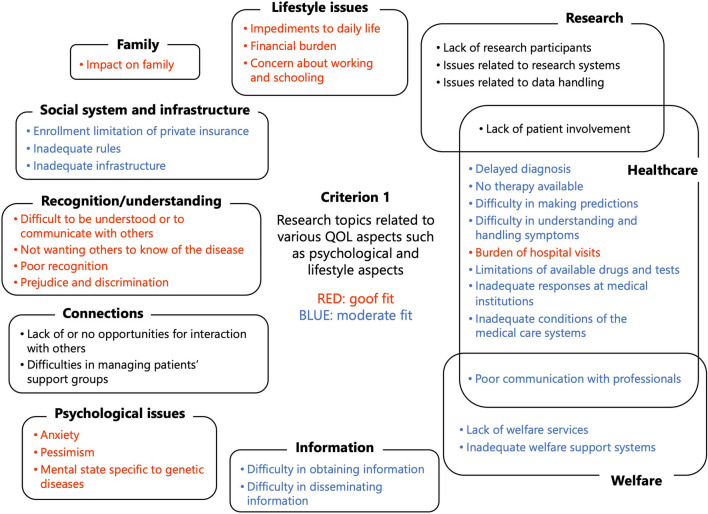
Results of the application of criterion 1, ‘research topics related to various QOL aspects, such as psychological and lifestyle aspects’

First, the results of applying criterion 1, ‘research topics related to various QOL aspects, such as psychological and lifestyle aspects’, led to the conclusion that the topics related to ‘lifestyle issues’, ‘recognition and understanding’, and ‘psychological issues’ had a good fit (Fig. 3).

**Fig. 4:**
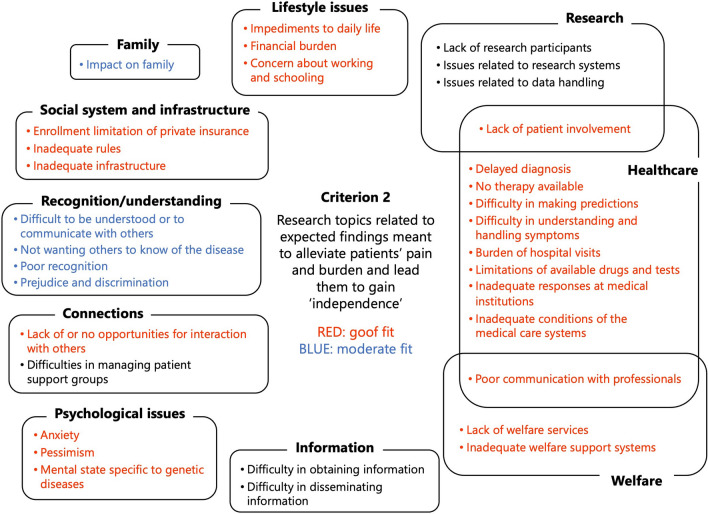
Results of the application of criterion 2, ‘research topics related to expected findings meant to alleviate patients’ pain and burden and lead them to gain “independence”’

During the application of criterion 2, ‘research topics related to expected findings meant to alleviate the pain and burden of the patients and lead them to gain “independence”’, the topics related to ‘healthcare’, ‘welfare’, ‘lifestyle issues’, ‘psychological issues’, and ‘social systems and infrastructure’ were identified to have a good fit (Fig. 4).

**Fig. 5:**
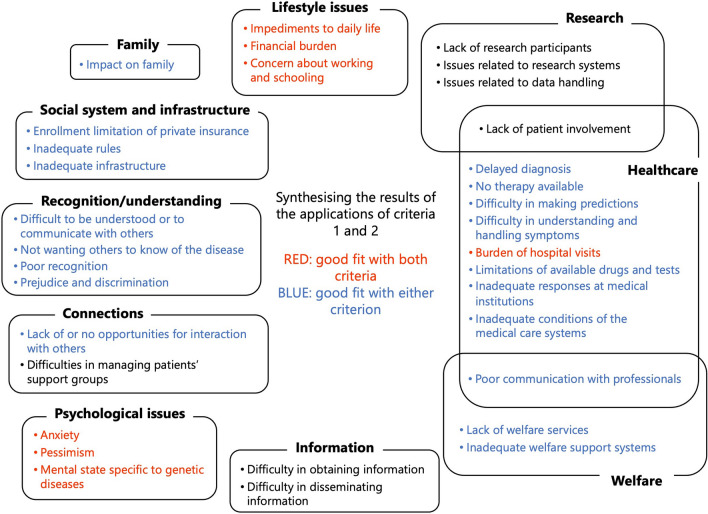
Synthesising the results of the applications of criteria 1 and 2

Because these criteria were particularly important for strengthening future research on rare diseases, the results of the application of the two criteria were synthesised. Consequently, seven research topics were identified as priority issues to be addressed, including ‘impediments to daily life’, ‘financial burden’, ‘anxiety’, and ‘burden of hospital visits’ (Fig. 5).

**Fig. 6:**
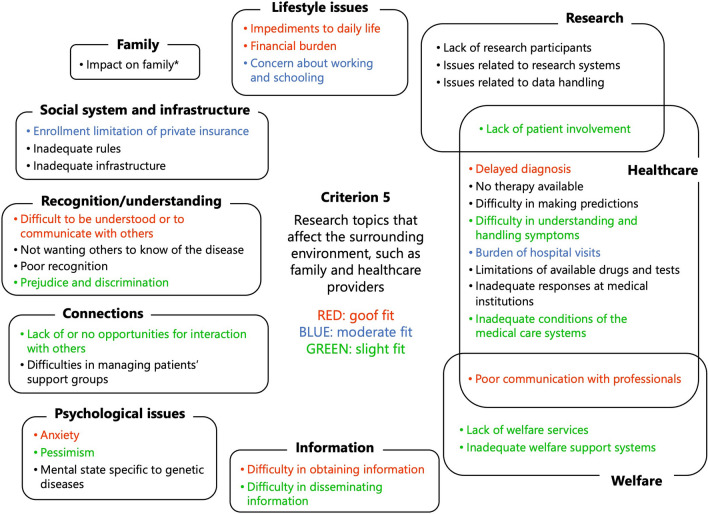
Results of the application of criterion 5, ‘research topics that affect the surrounding environment, such as family and healthcare providers. *When we tried to place the topic ‘impact on families’, we faced difficulty in deciding the relative importance of this topic in comparison to the other 32 topics, as it is, in effect, the criterion itself. Therefore, we decided not to list it as high priority.

Regarding criterion 5, ‘research topics that affect the surrounding environment, such as family and healthcare providers’, the following were newly identified as high-priority research topics, which had not previously been listed as such: ‘difficult to be understood or to communicate with others’, ‘difficulty obtaining information’, ‘poor communication with professionals’, and ‘delayed diagnosis’ (Fig. 6).

**Fig. 7:**
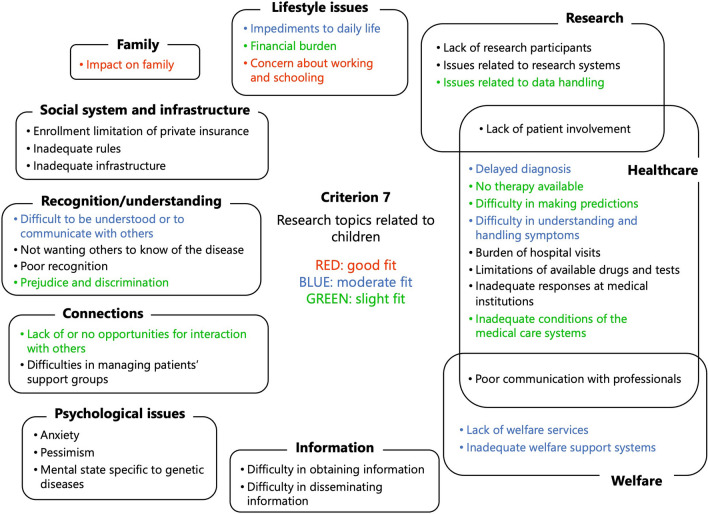
Results of the application of criterion 7, ‘research topics related to children’

**Fig. 8:**
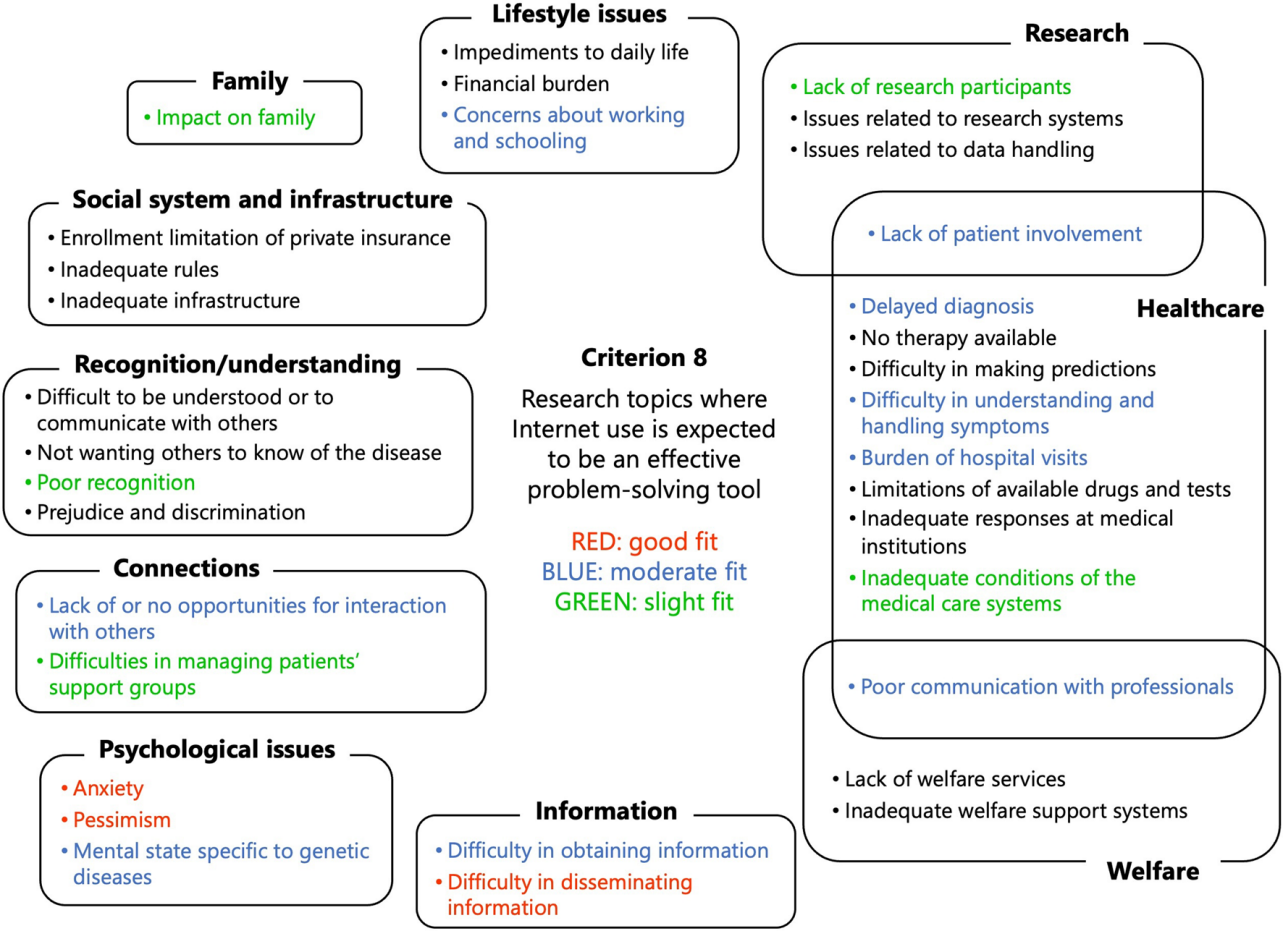
Results of the application of criterion 8, ‘research topics where Internet use is expected to be an effective problem-solving tool’

Concerning criterion 7, ‘research topics related to children’, and criterion 8, ‘research topics where Internet use is expected to be an effective problem-solving tool’, discussions were held in three separate groups, and the results were then consolidated. The research topics identified as a ‘good fit’ by all the groups were concluded as ‘good fit’ (indicated in red in the figures) in the ‘Commons’. According to criterion 7, ‘impacts on family’ and ‘concerns about working and schooling’ were indicated as being a ‘good fit’ (Fig. 7). By criterion 8, ‘anxiety’, ‘pessimism’, and ‘difficulty disseminating information’ were classified under ‘good fit’ (Fig. 8).

### Proposed research questions

Of the seven research topics identified as ‘particularly important research topics for strengthening future research on rare diseases’, by synthesising the results of the application of criteria 1 and 2 in Step 3, ‘impediments to daily life’ and ‘anxiety’ were selected for conducting brainstorming sessions on concrete research questions. Specific examples of the proposed research questions are presented in Box 3.

**Box 3:** Examples of the proposed research questions

Research questions on ‘anxiety’
· What are the characteristics of individuals who are more or less likely to experience anxiety?
· Are there any anxieties specific to Japan within the same disease area?
· Does knowing the social system changes one’s anxiety?
· When do patients with rare diseases have a sense of happiness?
Research questions on ‘impediments to daily life’
· What factors significantly reduce the QOL of patients and their families?
· What social systems would be needed to make it easier for patients to be able to go out?
· What do children find challenging in their school life?
· How well do healthcare providers understand patients’ daily life?
· How can impediments to daily life be classified?

## Discussion

This study aimed to create evidence that could be used to formulate and implement policies by constructing a space for continual deliberations among diverse stakeholders, including patients. In this section, we describe the creation of evidence and methods employed, based on our practical insights and understanding of future challenges. Additionally, we discuss the significance of the space created for deliberation in this study.

### Evidence for policy-making generated from the ‘Commons’

Through this study, we generated evidence with regard to two areas: ‘difficulties faced by patients with rare diseases’ and ‘priority research topics in the field of rare diseases’. First, this study revealed a wide range of difficulties faced by patients with rare diseases. This conforms to findings in existing literatures of shared experiences among patients with rare diseases [34–38]. However, topics such as ‘financial burden’, ‘enrolment limitation of private insurance’, ‘inadequate rules’, ‘inadequate infrastructure’, and ‘burden of hospital visits’ are newly presented in this study.

Second, we generated research topics through deliberation to prioritise the field of rare diseases. In particular, the seven research topics listed under the topic title ‘particularly important research topics for strengthening future research on rare diseases’—‘impediments to daily life’, ‘financial burden’, ‘concerns about working and schooling’, ‘anxiety’, ‘pessimism’, ‘mental state specific to genetic diseases’, and ‘burden of hospital visits’—point to desirable research topics that can improve understanding and help create strategies for resolving or reducing burden. Regarding these high-priority research topics, ‘financial burden’ and topics related to psychological issues are also in line with the results of studies reported in the existing literature [12, 47]. However, to the best of our knowledge, the presentation of the following topics as high priorities for research attention is something unique in our research results: ‘impediments to daily life’, ‘concern about working and schooling’, and ‘the burden of hospital visits’. Although the existing literature under the category ‘psycho-social impact’, covers these issues, the present study organises these problems in a different way than in the past, as stand-alone topics, which reflects patients’ perspectives. Moreover, while much of the existing literature lists items related to treatment, prevention, causes, and diagnosis as high priorities [12], none of these was included in the ‘particularly important research topics for strengthening future research on rare diseases’ presented in this study. This is partly related to the decision not to include the criterion, ‘research topics related to life and death’ as a priority research topic in this study on the basis that it has already been addressed with some degree of priority, demonstrating the need for the different perspectives that arise when the lived patient experience becomes a part of the research priority setting discussion.

The research topics were prioritised based on criteria valued more by patients and family members than by researchers and former policymakers (indicated in red in Fig. 6) so as to promote policies and research that prioritise patients’ perspectives. High-priority research topics identified according to criteria on specific important perspectives (shown in red in Figs. 7 and 8, respectively) serve as the basis for promoting research on topics that do not fall into the criterion of ‘particularly important research topics for strengthening future research of rare disease’, namely ‘impact on family’ and ‘difficulties disseminating information’. Thus, this study can optimise priority setting by selecting criteria tailored to the situation and needs of the individuals who will use the results of the priority setting.

Moreover, although the results are only exploratory, the proposed research questions on ‘impediments to daily life’ and ‘anxiety’ were novel concerns for some of the participating researchers, who normally focused on clinical issues and had never thought of them before. This is because the questions were based on the experiences of the patients and family members (i.e. demonstration of their expertise), and the ideas were freely conceived outside the academic field. Although we only proposed research questions as an exercise, the specific difficulties and perceptions of patients and family members were shared with the researchers (some of whom were also clinicians) through discussions on the subject, in a different way from the medical practice and previous workshops on this project. Consequently, researchers have gained significant new insights. To design actual studies in the future, refining the research questions through precise interactions between patients and researchers will be crucial. This is expected to result in novel findings. Furthermore, this process is time-consuming; the process itself leads to mutual learning, as discussed below.

### Methods to generate evidence for policymaking in this study

In this study, a space we called the ‘Evidence-Generating Commons’ was established to generate evidence for policymaking. The three main features of the methods are: (1) continual deliberations and co-creation among stakeholders, (2) examinations targeting multiple areas of rare diseases, and (3) outlining three steps for generating evidence.

#### (1) Continual deliberations and co-creations among stakeholders

Several initiatives are relevant to this study, including PSPs by the JLA, where research priorities are set by stakeholders, such as patients, their families, researchers, and policymakers [26]. In all cases, the time spent on generating results as a single project ranged from a few months to a year. Contrastingly, in this study, we conducted continual deliberations and co-creation for approximately three years. This is because one of our objectives was not to use existing methods, but to create a ‘space’ for generating evidence for policymaking, through practising specific activities by trial and error. Stated differently, patients and researchers created the process by examining the methods that can be used to generate evidence for policymaking in the field of medicine and healthcare. Hence, the method differs from the process of applying established methods for priority setting, such as PSPs.

Therefore, the participants could learn from one another about differences in ideas and perspectives from different standpoints, and trust was fostered through continual communication. Such mutual learning and trust-building contributed significantly to the deepening of discussions in the ‘Commons’. Specifically, during the initial steps, patients and family members took the lead in sorting out their difficulties. However, from the middle of the process, knowledge of ‘what research is’ was shared by the researchers and eventually compiled into an academic paper through the collaboration of both parties. This shows one way of co-creation through the complementary role of expertise drawn from patients’ experiences and researchers’ knowledge.

In developing this new set of methods, the role played by information and communication technology (ICT) is significant, enabling the creation of new communication space. Our ability to conduct over 20 workshops in total, with participants from all over the country, is undeniably a result of internet use. Participants could attend from their living rooms on weekday evenings after they finished work or household chores, or while looking after their children. It also helped busy researchers find time for workshops. The Internet is particularly useful when patients are spread out geographical, especially when organising frequent on-site workshops would be difficult [48]. Holding discussions so frequently likely contributed significantly to the fostering of trust, which is a prerequisite for co-creation.

#### (2) Examinations targeted at multiple rare disease areas

To date, most studies in the field of rare diseases have been conducted separately for each disease. The PSPs, which are typical examples of a research priority setting as described above, were also generally conducted targeting a single disease.

However, a lack of research resources has been pointed out as a major issue in the field of rare diseases [49, 50], in which cross-disease studies have recently attracted attention [51]. In this study, patients, family members, and researchers from several disease areas participated in a cross-disease study, and it became apparent that the difficulties faced by patients with rare diseases were often common regardless of the specific of the diseases being represented. Our results are supportive of the existing literature on the feasibility of priority setting targeted on multiple diseases [47, 52].

Previous studies have found that priority-setting results are specific to the health and daily life problems faced by patients with a specific disease [53]. However, the results of our priority setting were sufficiently acceptable for all participants with different rare diseases. Some research topics which are uniquely important to individual diseases may not be fully considered as high priority. However, setting a priority for all of the individual rare diseases separately would also be challenging. Therefore, we believe that priority setting for research on rare diseases focusing on multiple diseases would help identify necessary research topics that will positively impact larger numbers of patients. Additionally, patients’ participation in discussions on different diseases enabled them to express their opinion in a way that considered the situations in which patients with different diseases and their families were placed. The participants also could objectively observe diseases related to them, which led to new insights as well as clarification of the ‘characteristics of the disease’. This resulted in the participants’ learning.

#### (3) Outlining three steps for generating evidence

This study generated evidence in three steps: (i) clarification of difficulties faced by patients with rare diseases, (ii) development and selection of criteria for priority setting, and (iii) priority setting of research topics through the application of the criteria.

In particular, clarifying difficulties enabled us to discuss the issues as something more familiar and imaginable. A significant advantage of this method is the facilitation of agreement among stakeholders with different standpoints and circumstances by dividing priority setting into the steps of selecting and applying criteria rather than directly setting priorities.

However, during the discussions, the way of considering difficulties as research topics was not fully understood. In response to this, additional steps were taken to consider specific research questions. The primary purpose of this additional step was to enhance the patients’ understanding regarding the ‘research’. Indeed, the questions they created accurately represent the patients’ perspectives. Moreover, a short lecture by a researcher on ‘what research is’ was also given, which gradually deepened understanding and led to deeper discussions. Thus, when different stakeholders work together, ‘translating’ to bridge gaps in understanding and knowledge is important.

### The value of a ‘space’ for deliberations and co-creations among stakeholders

The process of generating evidence described above also resulted in the establishment of an unprecedented ‘space’ for deliberations and co-creations. Here, we discuss particularly the effects brought to the participants and the versatility of the ‘Commons’.

As described in the results, all participants of the ‘Commons’ experienced positive effects. These effects are in line with previous literature [54–57]. The project not only led to capacity building for all stakeholders but also created opportunities for interactions, when the participants talked about feelings that were not understood in their daily lives. This positively impacted them, and the ‘Commons’ became an important place for the participants. We are planning a different study to gain a deeper understanding of the participants’ experiences, which will be compiled in a separate report.

We also found the versatility of the ‘Commons’. In discussions in the ‘Commons’, as discussed above, opinions were sometimes expressed on measures to solve problems and policy proposals that go beyond setting priorities for research topics. This indicates a gap between the focus of this study on medical research and participants’ expectations of solutions to challenges. Conversely, this also suggests the versatility of the place of the ‘Commons’. We argue that this could be used as a method for deliberative democracy in the future; when stakeholders could be engaged to discuss how emerging technologies should be used in the field of medicine and healthcare.

### Discussion on the quality of the evidence generated in this study

**Fig. 9:**
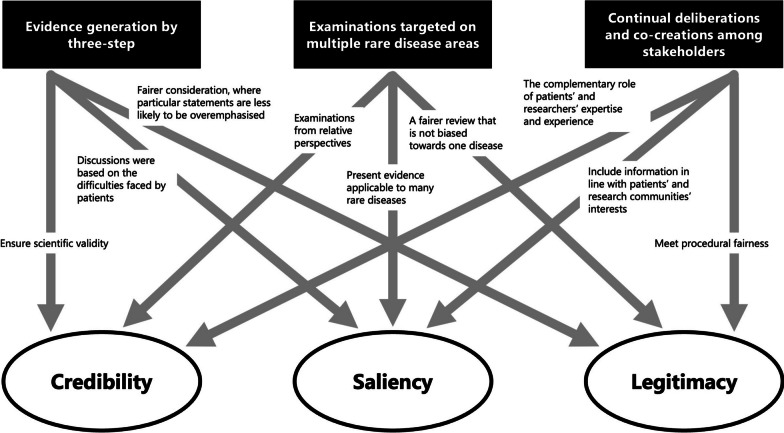
Impact of this study’s methods of generating evidence on the nature of the evidence generated

Finally, we discuss the results from a theoretical perspective. We particularly focused on how the methods of generating evidence in this study affected the nature of the evidence generated, in terms of the three attributes of evidence—credibility, saliency, and legitimacy [21, 22]—in policymaking as suggested by Cash et al. and Parkhurst as described above. An overview of this process is shown in Fig. 9.

First, the scientific validity or reliability of the evidence is ensured through a systematic method in three steps. The first step is to clarify patients’ difficulties as a starting point for discussion. Additionally, saliency is enhanced because it includes appropriate information for decision-makers and the audience. Furthermore, the two-step approach to prioritise the development and selection of criteria and the application to set priorities ensures legitimacy by contributing to a fair process in which specific statements do not overly influence the outcome.

Second, targeting multiple rare diseases helps to contribute to the credibility of evidence by encouraging relative perspectives. Further, evidence that can be applied to many diseases is more salient for policymakers, especially in national rare-disease policies, because those that cover only certain diseases are considered inappropriate. Simultaneously, cross-disease examinations also improve the legitimacy of the evidence as a fairer consideration that is not biased towards one disease.

Third, we would argue that continual deliberations and co-creation among stakeholders can generate reliable evidence through the complementary role of the expertise of patients and their families based on their experiences and researchers’ expertise. Moreover, patients’ participation with researchers in the process of generating evidence on various diseases indicates that the generated information conforms to the interests of patients and patient groups facing various difficulties presented in this study, as well as the interests of researchers seeking solutions through research. Furthermore, different stakeholders, such as patients, family members, and researchers from multiple rare-disease areas, were involved in the priority-setting discussions, and more than 20 workshops were held repeatedly to ensure procedural justice.

In terms of salience, current policymakers’ non-participation in the ‘Commons’ may result in insufficient salience for them at present, although former policymakers who participated significantly contributed towards ensuring that the evidence was relevant for policymaking. In response to this, our strategy was not for current policymakers to participate in discussions in the ‘Commons’ from the outset, but to engage in dialogue with them based on information generated from the discussions in the ‘Commons’, and then compile it as final evidence. The strength of this strategy is that it has generated evidence that is more tailored to the needs of patients and researchers and not bounded by existing policy. Conversely, while the possibility of a direct link to policy implementation increases when the study is led by the government, drawbacks also exist, such as directing the study content to some degree and considering consistency with existing policy. Involving current policymakers while maintaining the strengths of this strategy remains a challenge. Certainly, for information generated from discussions in the ‘Commons’ to be demonstrated as effective evidence for policy, it should be referred to in policymaking, although in reality, it is only demonstrated by its implementation in policy. Presently, while information has not yet been demonstrated in the policy field as such, based on Cash’s argument, the three attributes are met and could be considered ‘evidence’.

In summary, the quality of evidence generated by this study was strengthened from multiple aspects. A critical point here is that our approach would enhance EIPM by presenting methodologies for involving patients, researchers, and former policymakers in the policy making process and by fostering knowledge sharing among different stakeholders and consulting target groups to get their perspectives [58].

Furthermore, information produced by stakeholders can be important and valuable not only for policy-making activities but also for a range of activities undertaken by respective stakeholders. For example, important decisions within the respective organisations, such as research funding by patient associations or the setting of research topics by researchers, could be better directed if they are evidenced and reasoned by information that is credible, salient, and legitimate.

### Limitations of this study

The main limitation of this study was the small number of target diseases and participants. The study ultimately targeted 10 rare hereditary diseases without curative treatment, and which carry a long-term disease burden. However, all the participants were in a condition and environment that allowed them to participate in the workshops and discuss their issues. Consequently, the views on diseases held by patients who could not participate in such discussions for various reasons were not adequately reflected. However, this does not mean such views are not reflected at all, because family members of patients with rare diseases, such as those with childhood-onset and cognitive impairment, participated in the workshops instead. It was particularly demonstrated that even in the case where patients found it difficult to participate directly in the discussion, their families could participate in reflections and deliberations regarding the patients’ opinions. Furthermore, as this study was conducted using ICT, the impact of the non-participation of those who could not participate in ICT-based settings for reasons such as ICT illiteracy on the results of this study needs to be examined separately.

## Conclusion

This paper reports on the Commons Project’s approach to generating evidence for rare-disease policies and effective stakeholder involvement for this purpose. The method was characterised by a systematic three-step process, cross-disease examinations, continual deliberations and co-creation among stakeholders, and taking difficulties faced by patients with rare diseases as a starting point to set research priorities in the field of rare diseases. The quality of these processed as evidence for policymaking has been discussed from a theoretical perspective, but policy implementation is still not complete. Most emphatically, positive effects such as capacity building, creation of opportunities for interactions with others, mutual understanding, and empathy were brought to all the ‘Commons’ participants throughout the process—the continual multi-step work as a research team beyond expectations at the time of project planning.

In Japan, patient-led initiatives have become increasingly visible and had influences on policy making in recent years, including the passage of the Intractable Disease Law, as mentioned above [40, 41]. Simultaneously, national organisations such as AMED and PMDA are promoting patient and public involvement in medical research, and in the development and approval review of drugs [10, 11]. Patients are expected to collaborate with researchers as partners. Despite the expectations of both patients and policymakers, translating patients’ views and perspectives into actual policy is not easy. To our knowledge, few examples of deliberations and co-creation between patients and professionals as partners exist. In response to this situation, this study provides insight into the promotion of patients and the public’s involvement, by realising this social trend academically and scientifically.

We will begin with full-fledged discussions with policymakers regarding policy implementation and plan to examine the participants’ experiences in this project.

### Supplementary Information

**Additional file 1.** Programme of the on-site workshop.

## Data Availability

The datasets used and/or analysed during the current study are available from the corresponding author upon reasonable request.

## Abbreviations

AMED: Agency for Medical Research and Development
CHNRI: Child Health and Nutrition Research Initiative
EBM: Evidence-based medicine
EIPM: Evidence-informed policymaking
ICT: Information and communication technology
JLA: James Lind Alliance
PMDA: Pharmaceuticals and Medical Devices Agency
PSP: Priority Setting Partnership
QOL: Quality of life
RCT: Randomised controlled trials
