# Supplementary material for: Enhancing evidence-informed policymaking in medicine and healthcare: stakeholder involvement in the Commons Project for rare diseases in Japan
Source: Res Involv Engagem. 2023 Nov 29;9:107. doi: 10.1186/s40900-023-00515-5 (PMC10685685; doi:10.1186/s40900-023-00515-5)
Supplement: Supplementary file 1 — Additional file 1. Programme of the on-site workshop. [file 40900_2023_515_MOESM1_ESM.docx]

Supplementary file 1. Programme of the on-site workshop

| 13:30 - 13:35 | Opening remarks |
| --- | --- |
| 13:35 - 13:40 | Briefing about the Commons Project |
| 13:40 - 13:50 | Explanation on today’s work |
| 13:50 - 13:55 | Self-introduction (in group) |
| 13:55 - 14:05 | Writing out challenges and proposals (individual work 1) |
| 14:05 - 14:45 | Sharing and organising individual sticky notes (group work 1) |
| 14:45 - 15:00 | Break |
| 15:00 - 15:05 | Writing down additional suggestions (individual work 2) |
| 15:05 - 15:20 | Sharing and organisation of additional suggestions and organising sticky notes on sheets (group work 2) |
| 15:20 - 16:10 | Presentations by each group |
| 16:10 - 16:30 | Reflection |

First, individual participants had ten minutes to write down difficulties on yellow sticky notes and solutions on red sticky notes, and the next 20 minutes were used for group work to collect and organise sticky notes with similar content. Next, the individual participants were asked to write down the solutions they produced following the previous group’s work on blue sticky notes again in five minutes, and finally, the group organised and discussed them once more.
